# Correlation study of LINC02609 and SNHG17 as prognostic biomarkers of kidney renal clear cell carcinoma and therapeutic sensitivity based on public data and *In Vitro* analysis

**DOI:** 10.3389/fimmu.2025.1592474

**Published:** 2025-05-26

**Authors:** Chaoqun Xing, Weiwei Zou, Yangqin Li, Ti Zhang, Fan Yao, Zhi-Yong Yao, Xiao-Liang Xing

**Affiliations:** ^1^ The First Affiliated Hospital of Hunan Medical University, School of Public Health and Emergency Response, Hunan University of Medicine, Huaihua, Hunan, China; ^2^ Gynecological Oncology Department, The Second People’s Hospital of Huaihua, Huaihua, Hunan, China

**Keywords:** KIRC, cupropsis, lncRNAs, prognosis, immunotherapy, chemotherapy

## Abstract

**Background:**

Cuprotosis, a newly identified form of regulated cell death, has emerged as a potential therapeutic target for cancers. Kidney renal clear cell carcinoma (KIRC) is frequently metastatic at diagnosis, resulting in poor prognosis. This study aimed to identify prognostic biomarkers and construct a risk model to improve survival prediction and guide therapeutic strategies for KIRC patients.

**Methods:**

Differential expression analysis, Cox regression, and risk modeling were performed using transcriptomic and clinical data. The response to immunotherapy and the sensitivity to chemotherapy drugs were analyzed through the Tumor Immune Dysfunction and Exclusion (TIDE) database and the Genomics of Drug Sensitivity in Cancer2 (GDSC2) database. Functional validation of LINC02609 was conducted in renal carcinoma A498 cells using siRNA-mediated knockdown.

**Results:**

LINC02609 and SNHG17 were significantly upregulated in KIRC tissues and independently associated with poor overall survival. The risk model constructed using those two candidate biomarkers (LINC02609 and SNHG17) exhibited high predictive accuracy as measured by the value of area under the curve (AUC). Immune status analysis showed that high- and low-risk KIRC patients exhibited abnormalities immune landscapes. TIDE analysis suggested that the risk model was significantly correlated with multiple immunotherapy-related signatures. RNA-sequencing (RNA-seq) analysis indicated that inhibition of LINC02609 would lead to abnormal activation of the mitogen-activated protein kinases (MAPK) signaling pathway. *In vitro* experiments confirmed that LINC02609 knockout inhibits the proliferation, migration, and invasion of A498 cells by suppressing the MAPK signaling pathway.

**Conclusion:**

The candidate biomarker LINC02609 regulates the progression of renal cell carcinoma through the MAPK signaling pathway. The risk model constructed using LINC02609 and SNHG17 was significantly correlated with multiple immunotherapy-related signatures, suggesting that it might be used for the determination of immunotherapy options in KIRC

## Introduction

Kidney cancer ranks among the most prevalent malignancies in the genitourinary system, accounting for approximately 3% of global cancer diagnoses (1). According to 2021 epidemiological statistics, it caused over 400,000 new cases (2.2% of all cancers) and 180,000 cancer-related deaths worldwide (2). Among renal cell carcinoma subtypes, kidney renal clear cell carcinoma (KIRC) predominates, comprising 85% of cases and demonstrating aggressive clinical behavior (3, 4). Notably, a substantial subset of KIRC patients (25–30%) present with metastatic disease at initial diagnosis, severely limiting therapeutic options (5). Furthermore, even after surgical resection, the postoperative recurrence rate remains strikingly high (30–50% in advanced stages), culminating in a dismal 5-year survival rate of approximately 10% (6, 7). These persistent clinical challenges highlight the critical need for developing reliable prognostic stratification tools to optimize personalized therapeutic decision-making.

Copper ions, essential cofactors for enzymatic redox reactions, are tightly regulated under physiological conditions to maintain cellular homeostasis (8). Dysregulated copper accumulation triggers metabolic dysfunction and induces a novel copper-dependent cell death mechanism termed cuproptosis (9–11). This process is mechanistically distinct from other forms of cell death, characterized by mitochondrial respiratory collapse via aggregation of lipoylated tricarboxylic acid (TCA) cycle proteins (12). Emerging studies link cuproptosis dysregulation to multiple malignancies, including lung, prostate, breast, gastric, and thyroid cancers (13–17). For example, Shen et al. reported that the copper ionophore disulfiram induces immunogenic cell death, enhances dendritic cell activation and T-cell infiltration, and synergizes with programmed cell death protein 1 (PD-1) inhibitors to suppress tumor growth in preclinical models (18). Similarly, Mao et al. identified that the cuproptosis-related gene metal responsive transcription factor 1 (MTF1) inhibits KIRC progression by suppressing tumor proliferation and modulating immune cell infiltration (19). Notably, the ferroptosis and cuproptosis-related protein ferredoxin 1 (FDX1) regulates lipoylated protein metabolism and drives cancer progression and metastasis in diverse malignancies (20, 21). Additionally, copper chelators have been shown to reverse cisplatin resistance through modulation of mitochondrial copper dynamics (22). Intriguingly, the cuproptosis-associated protein cyclin dependent kinase inhibitor 2A (CDKN2A) has been implicated in cellular sensitivity to copper-mediated proliferation (12). Previous studies indicated that dysregulation of cuproptosis-related pathways may promote oncogenesis via perturbation of key signaling cascades, including the MAPK and phosphatidylinositol 3-kinase (PI3K)- protein kinase B (PKB, AKT) pathways (23–25).

Long non-coding RNAs (lncRNAs), defined as transcripts exceeding 200 nucleotides in length with no protein-coding capacity, serve as critical regulators of tumorigenesis and cancer progression through epigenetic modulation, transcriptional regulation, and post-transcriptional modifications (26–33). Building upon the emerging role of cuproptosis in KIRC pathogenesis, this study performed an integrative multi-omics analysis to identify prognostic lncRNA biomarkers associated with cuproptosis, construct a risk stratification model, and systematically evaluate its clinical utility for prognosis prediction and therapeutic optimization in KIRC. Furthermore, functional validation experiments were conducted to elucidate the mechanistic contributions of candidate lncRNAs to tumor progression, providing novel therapeutic targets for KIRC management.

## Materials and methods

The flow chart in **Supplementary Figure S1** depicted the data analysis process.

### Data collection and preprocessing

RNA-sequencing (RNA-seq) raw count data and corresponding clinical metadata were retrieved from the Cancer Genome Atlas (TCGA) and International Cancer Genome Consortium (ICGC) databases, comprising 602 samples (72 normal vs. 530 KIRC) from TCGA and 136 samples (45 normal vs. 91 KIRC) from ICGC (**Table 1**). The TCGA-KIRC cohort was designated as the discovery/training cohort, while the ICGC-KIRC cohort served as the independent validation cohort. The combined dataset (TCGA + ICGC) constituted the entire cohort for pooled analyses. A curated set of 16,901 annotated lncRNAs was obtained from the GENCODE database (version 38). Nineteen cuproptosis-related genes (CRGs) were selected based on prior mechanistic studies (12, 34), including: *ATP7A*, *ATP7B*, *CDKN2A*, *DBT*, *DLAT*, *DLD*, *DLST*, *FDX1*, *GCSH*, *GLS*, *LIAS*, *LIPT1*, *LIPT2*, *MTF1*, *NFE2L2*, *NLRP3*, *PDHA1*, *PDHB*, and *SLC31A1*. The DESeq2 package (v1.26.0) in R (v3.6.1) was employed to identify differentially expressed genes (DEGs) with the following thresholds: baseline expression ≥ 100 counts (baseMean), absolute log2 fold change ≥ 1, and adjusted p-value (padj) < 0.05.

**Table 1:** Clinical information of KIRC patients in different group.

Clinical characteristics		Training (N=530)	Validation (N=91)	Entire (N=621)
Vital	Alive	357	61	418
	Dead	173	30	203
M	M0	420	81	501
	M1	78	9	87
	MX	32	1	33
N	N0	239	79	318
	N1	16	2	18
	NX	275	10	285
T	T1	271	54	325
	T2	69	13	82
	T3	179	22	201
	T4	11	2	13

### Single-cell sequencing analysis

The single-cell sequencing data (GSE121636) were collected from the Gene Expression Omnibus (GEO) database. Seurat package in R (3.6.1) was applied for KIRC data integration and quality control (35). Vlnplot, Dimplot, and Featureplot package in R (3.6.1) were used to visual genes expression. The FindClusters function is used to group cells together. The tumor cells were identified by intercnv packets and copycat packets in R (3.6.1). The visualization of dimensionality reduction is realized by tSNE function with default parameter.

### Immune profile analysis

Estimation of STromal and Immune cells in MAlignant Tumor tissues using Expression data (ESTIMATE) algorithm in R (3.6.1) was used to evaluate the tumor microenvironment (TME) using all genes normalized expression data (36). Single sample Gene Set Enrichment Analysis (ssGSEA) algorithm in R (3.6.1) was used to evaluate the immune score of immune factors and cells using all genes normalized expression data (37).

#### Cox regression analysis

The median expressed value of each gene was used to divide the patients with KIRC into low and high expression group. The univariate Cox regression analysis was used to screen the overall survival (OS) related signatures followed with least absolute shrinkage and selection operator (LASSO) analysis in all KIRC patients. Multivariate Cox regression analysis was carried out for overall survival related biomarkers to screen the potential biomarkers.

#### Risk model construction

After multivariate Cox regression analysis, the potential signatures were used to construct the prognosis model by the following formula: $\text{Risk socre}=\sum_{\text{i}=1}^{\text{n}}\text{β}\text{(Xi)}*\text{Exp}(\text{Xi})$ (38). To conduct integrated analysis of different samples, we built the comprehensive-index (C-index). C-index = (Risk score – Min)/Max. The Youden index was used as the optimal cut-off value to regroup the patients with KIRC into low and high-risk group.

#### Sensitivity analysis

Differentially expressed genes between high- and low-risk KIRC patients were identified by DESeq2 algorithm in R (3.6.1) and then used to evaluate chemotherapy sensitivity by oncoPredict algorithm, and immunotherapy sensitivity by Tumor Immune Dysfunction and Exclusion (TIDE) database (http://tide.dfci.harvard.edu/login/).

### Cell culture and transfection

HK2, A498, CAKI-1, and 786-O cells were obtained from the American Type Culture Collection (ATCC), and maintained in MEM + 10% FBS +1% PS, MEM + 10% FBS +1% PS, McCoy’s 5a + 10% FBS +1% PS, and RPMI1640 + 10%FBS+1% PS respectively. All cells were authenticated and tested for mycoplasma contamination, and were maintained at 37 °C in an incubator containing 5% CO2.

LINC02609‐specific siRNAs were synthesized by GENERAL BIOL (China). Si-NC: 5′‐UUCUCCGAACGUGUCACUUTT‐3′. Si-RNA1: 5’-GAGAGAAGAGCAUGAUGAATT-3’. Si-RNA2: 5’-GGUCAAUGCAUGUACUUAATT-3’.

SiRNAs were transfected into cells using Lipofectamine 2000 Reagent (Invitrogen, USA) according to the manufacturer’s instructions. MAPK agonist (Ro 67–7476) was obtained from MedChemExpress.

#### Quantitative reverse transcription–PCR

Total RNA from different cells were extracted using Trizol reagent (Life technologies, NY, USA) according to the manufacturer’s instruction. 2.0 μg of total RNA were reverse-transcribed using the RevertAid First Strand cDNA Synthesis Kit (Thermo Fisher, Waltham mass, USA). The mRNA levels were examined with qPCR using 1 × SYBR Green PCR master mix (Thermo Fisher, Waltham mass, USA) by a C1000 touch Thermal Cycler. The primers sequence used in our present study was showed as following (**Table 2**).

**Table 2:** Primer sequence information.

LncRNAs	Forward Primer	Reverse Primer
LINC02609	CAGCGCCCGTTTATTTGAG	AGTGCTCCTGGCTTCTTCTTGTA
SNHG17	TGGGAGTGTCACATGACTGC	TGGGAGTGTCACATGACTGC
PRKAR1B-AS2	TTGGACACTGCCCATCTTCC	TGCAGCCACGGGATGTTTAT
U6	CTCGCTTCGGCAGCACA	AACGCTTCACGAATTTGCGT

#### Cell counting kit-8 analysis

Cell proliferation was assessed using the cell counting kit‐8 (CCK‐8) assay (Beyotime Technology, China). 24h, 48h, 72h after transfection, the absorbance was measured by adding 10 μL CCK-8 reagent per well for 2 h.

#### Wound healing assay

Cell migration was assessed using wound healing assay. When the cell fusion rate of the six-well plate was about 90%, the vertical scraping was performed with a 1ml straw head. The suspended cells were cleaned with PBS and treated with interference. Serum-free medium was cultured for 24h in a 37°C-incubator containing 5% CO_2_.

#### Invasion assay

The cells were implanted in a 6-well plate and cultured in a medium containing 10% fetal bovine serum and penicillin-streptomycin in 37°C-incubator containing 5% CO2. After 48 weeks, the colonies were fixed with 100% methanol for 30 minutes, and then stained with 0.5% crystal violet solution. The colonies were rinsed with clean water and counted under a microscope.

#### Western blot and antibody

The cells were homogenized in 2 × SDS gel-loading buffer (50 mM Tris–HCl at pH 6.8, 2% SDS and 10% glycerol) with 1 × Protein inhibitor cocktail. Proteins were resolved by SDS–PAGE and transferred onto PVDF membranes. The PVDF membranes was blocked in 5% skim milk/Tris-buffered saline that contained 0.1% Tween 20, incubated with the primary antibodies at 4°C overnight, and incubated with second antibody. The PVDF membranes were visualized with enhanced chemiluminescence western blotting detection reagents. The antibodies were listed as following: Phospho-ERK (T202/Y204) (Abways, China), ERK (T185/Y187) (Abways, China).

### RNA-sequencing

Total RNA was extracted with TRIzol reagent following the recommendations of the manufacturer. RNA was sequenced using Illumina platform in Beijing Qingke Biotechnology Co., LTD. The sequencing depth for each sample was >20 million reads. The reads were aligned with TopHat 2.0.13 to GRCh38.112 with default parameters. Transcript abundance was measured in fragments per kb of exon per million fragments mapped (FPKM).

### Statistical analysis

A repeated-measure ANOVA followed by Bonferroni *post hoc* tests or unpaired two-tail Student’s *t test* was used as indicated.

## Results

### Screening of CRDELs as a prognostic marker of KIRC

To determine whether cuprotosis differed among KIRC patients, we estimated the cuprotosis score for each sample using cuprotosis related genes. Analysis of differences from the TCGA and ICGC datasets showed that cuprotosis scores were significantly higher in both KIRC patients (**Supplementary Figures S2a, b**). Therefore, to obtain suitable cuprotosis-related lncRNAs as biomarkers, we first performed differential expression analysis. There were significant differences in 3879 genes between the normal and KIRC patients (**Supplementary Figure S2c**). Of these, 361 (278 up regulated and 83 down regulated) were differentially expressed lncRNAs (DELs) (**Supplementary Figure S2d**). Next, 361 DELs were used for Pearson correlation analysis with cuprotosis-related genes. Among them, 270 DELs were significantly correlated with 19 cuprotosis-related genes (**Supplementary Figures S2e-j**). Therefore, these 270 DELs are named as cuprotosis-related DELs (CRDELs). Before the biomarker screening, we conducted consensus analysis. The results showed that these 270 CRDELs could well divide KIRC patients into two different groups, and the overall survival of KIRC patients between the two groups was significantly different (**Supplementary Figures S2k, l**).

**Figure 1:**
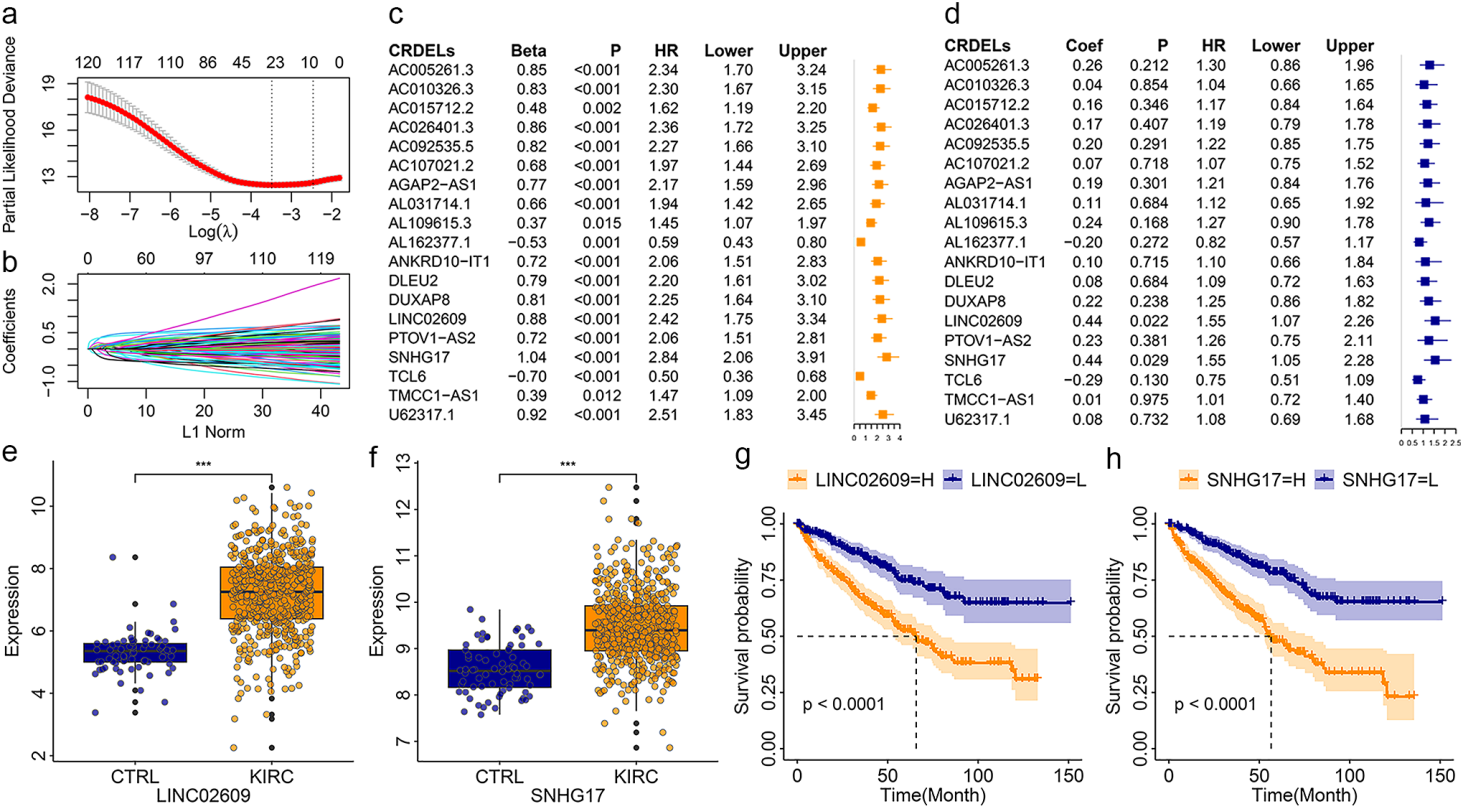
Identification of CRDELs as candidate biomarkers. **(a)** The LASSO tuning parameters. **(b)** The CRDELs LASSO coefficient profile. **(c, d)** Forest map of overall survival related CRDELs verified by univariate **(c)** and multivariate **(d)** Cox regression analysis. **(e, f)** Expression LINC02609 **(e)** and SNHG17. **(g, h)** K-M curve of LINC02609 **(g)** and SNHG17 **(h)**. *P<0.05. **P<0.01. ***P<0.001. NS, no significance.

Analysis of differences showed that 150 of 270 CRDELs were significantly different between surviving and deceased KIRC patients. Univariate Cox regression analysis was carried out for these 150 DELs followed by LASSO analysis (**Figures 1a, b**). The results showed that 23 CRDELs were significantly associated with the OS of KIRC (**Figure 1c**). Four CRDELs (**Supplementary Figures S3a-d**) were significantly elevated in KIRC patients. However, Kaplan-Meier (K-M) curves showed that KIRC patients with high expression showed better survival (**Supplementary Figures S3e-h**). Therefore, in the subsequent analysis, we only performed multivariate Cox regression analysis for those 19 CRDELs (**Figure 1d**). The results showed that LINC02069 and SNHG17 were independently correlated with the OS of KIRC. Their expression and its relationship to the OS is shown in **Figures 1e-h**.

### Construction and validation of risk models

**Figure 2:**
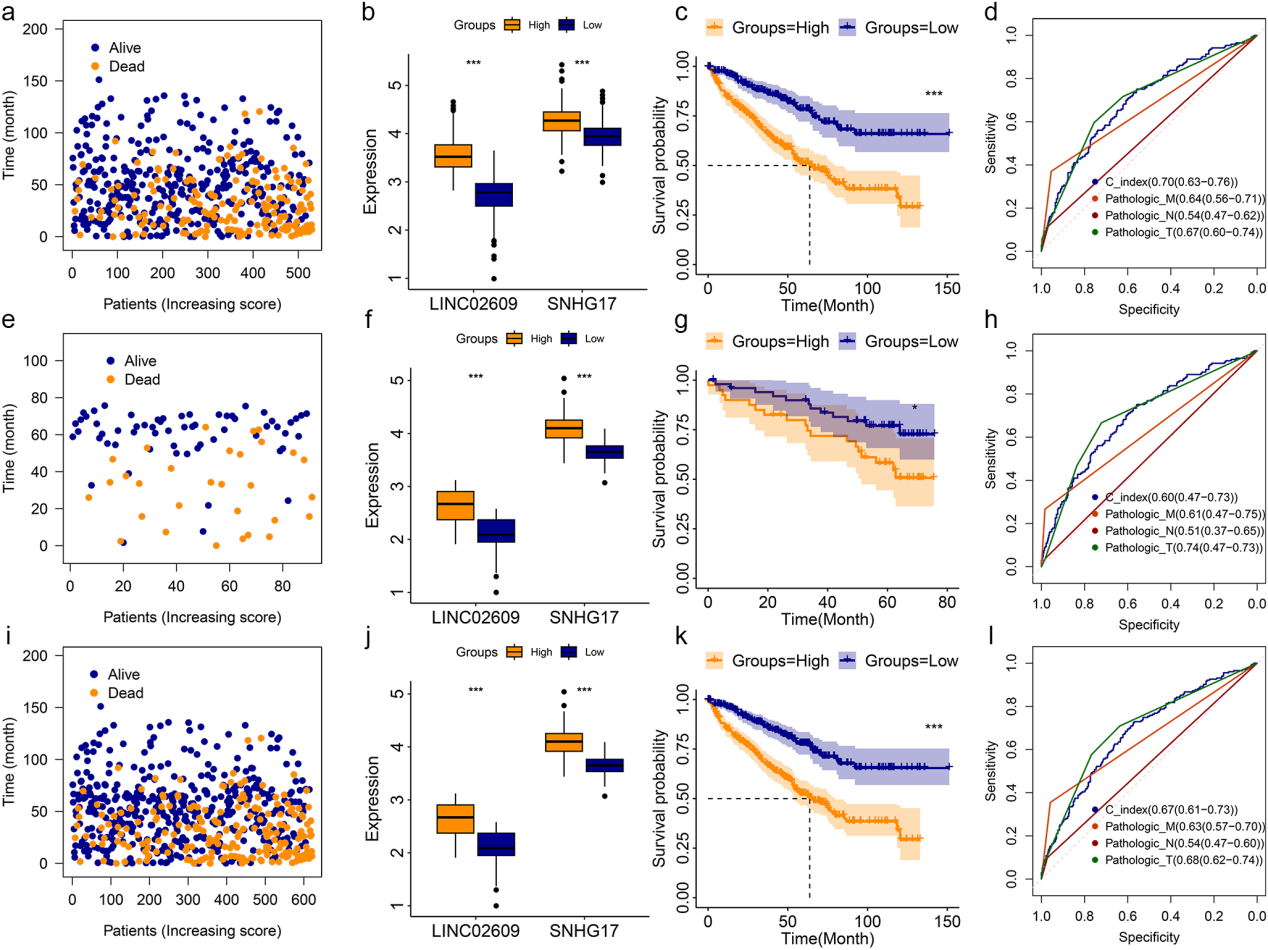
Construction and validation of risk model base on TCGA and ICGC database. **(a-d)** The survival status **(a)**, expression status **(b)**, K-M curve **(c)**, and ROC curve **(d)** of risk model in TCGA training group. **(e-h)** The survival status **(e)**, expression status **(f)**, K-M curve **(g)**, and ROC curve **(h)** of risk model in TCGA training group. **(i-l)** The survival status **(i)**, expression status **(j)**, K-M curve **(k)**, and ROC curve **(l)** of risk model in TCGA training group. *P<0.05. **P<0.01. ***P<0.001. NS, no significance.

Multivariate Cox regression analysis showed that LINC02609 and SNHG17 were independently related to KIRC OS. Therefore, LINC02609 and SNHG17 were used to construct a risk model. With the increase of risk value, the survival time of KIRC patients showed a decreasing trend (**Figure 2a**). The expression of LINC02609 and SNHG17 was not only highly expressed in KIRC patients, but also significantly increased in high-risk KIRC patients (**Figure 2b**). The K-M curve shows that high-risk KIRC patients exhibit significantly poorer OS status (**Figure 2c**). To evaluate the accuracy of the risk model for prognostic evaluation, we plotted the ROC curve. The results showed that the AUC value of the risk model was 0.70, which was slightly higher than that of pathologic M, pathologic N and pathologic T (**Figure 2d**).

To further validate the feasibility of the risk model, KIRC data from the ICGC were used as other independent samples for validation analysis. Similar results were obtained in the analysis of this independently verified sample (**Figures 2e-h**). In addition, we combined KIRC data from TCGA and ICGC and obtained similar results (**Figures 2i-l**). In the training group, validation group, and entire group, we found that high-risk KIRC patients showed significantly poorer OS (**Figures 2c, g, k**). They all had AUC values above 0.60 (**Figures 2d, h, l**).

### Correlation analysis for risk models with different clinical phenotypes

In the entire group, we created nomograms to understand the relationship between the risk model and different clinical features. It showed the status of one of the KIRC patients in different clinical phenotypes and risk models (**Supplementary Figure S4a**). The results show that the KIRC predicted by the risk model at 1-, 3-, and 5- years has a high agreement with the real structure (**Supplementary Figure S4b**). The ROC curve showed that the risk model had high accuracy in 1-, 3- and 5-year KIRC predictions. Their AUC values were 0.77,0.69 and 0.68, respectively (**Supplementary Figure S4c**).

**Figure 3:**
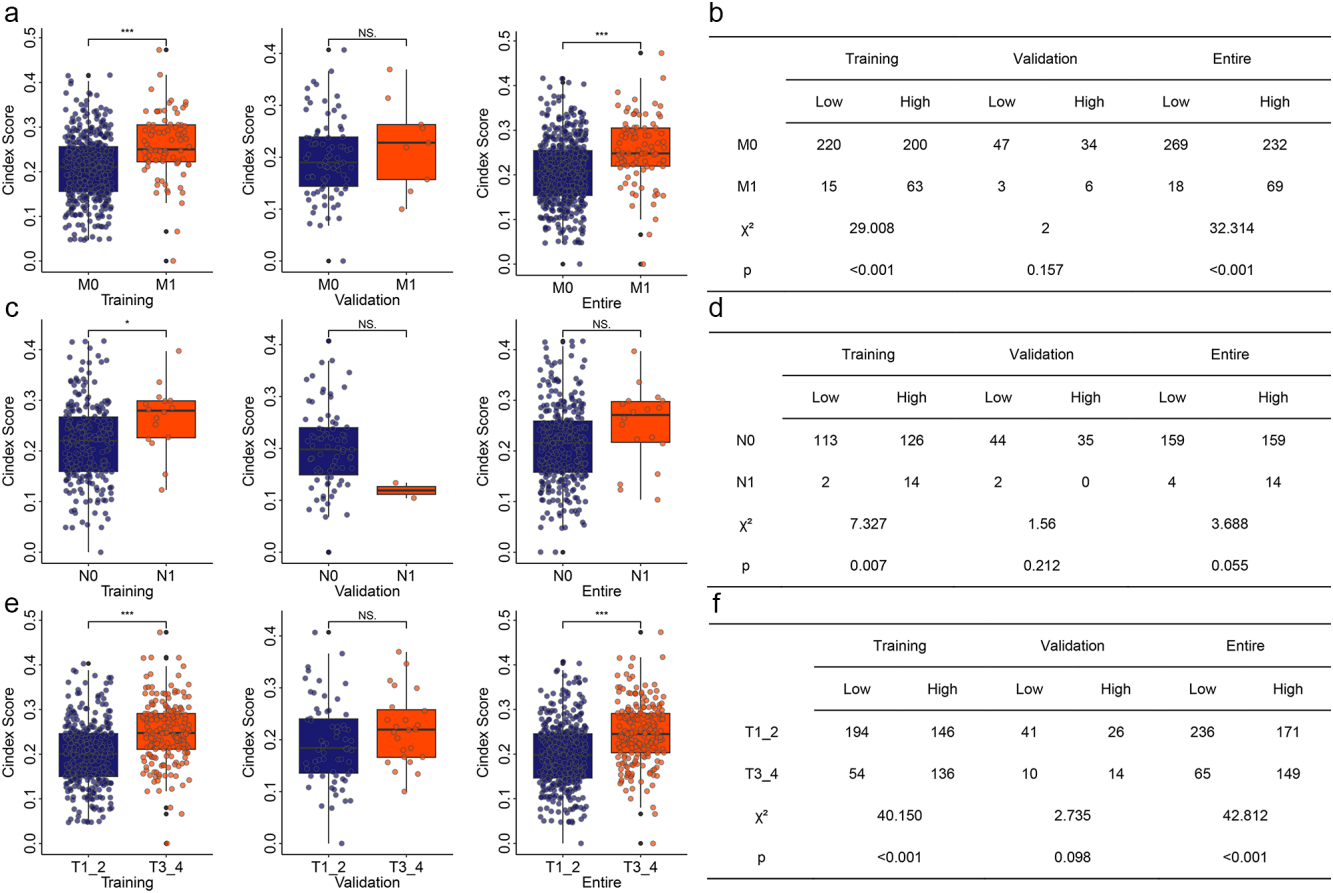
Correlation of risk model with different clinical features. **(a)** Correlation of risk model with pathologic M in different group. **(b)** Chi-square analysis for risk model and pathologic M. **(c)** Correlation of risk model with pathologic N in different group. **(d)** Chi-square analysis for risk model and pathologic N. **(e)** Correlation of risk model with pathologic T in different group. **(f)** Chi-square analysis for risk model and pathologic T. *P<0.05. **P<0.01. ***P<0.001. NS, no significance.

Most KIRC patients already have metastatic cancer when they are first diagnosed. Even after surgery, KIRC has a high recurrence rate. These may be the reasons leading to the poor prognosis of KIRC. Therefore, we explored the relationship between risk models and different clinical features. In the training group, KIRC patients with pathologic M1 had significantly higher risk value than those KIRC patients with pathologic M0 (**Figure 3a**). In the validation group, KIRC patients with pathologic M1 showed an increase risk value, but there was no significant difference (**Figure 3a**). When we performed our combined analysis, KIRC patients pathologic M1 still had significantly higher risk values than KIRC patients with pathologic M0 (**Figure 3a**). Based on pathologic N, the risk value of KIRC with pathologic N1 was significantly higher in the training group than in KIRC patients with pathologic N0 (**Figure 3c**). Based on pathological T, we obtained similar results to cases based on pathological M (**Figure 3c**). In both the training and entire groups, the risk values for KIRC with pathologic T1+T2 were significantly higher than those for KIRC with pathologic T3+T4 (**Figure 3e**). Chi-square analysis showed similar results (**Figures 3b, d, f**).

### Sensitivity analysis of immunotherapy and chemotherapy

**Figure 4:**
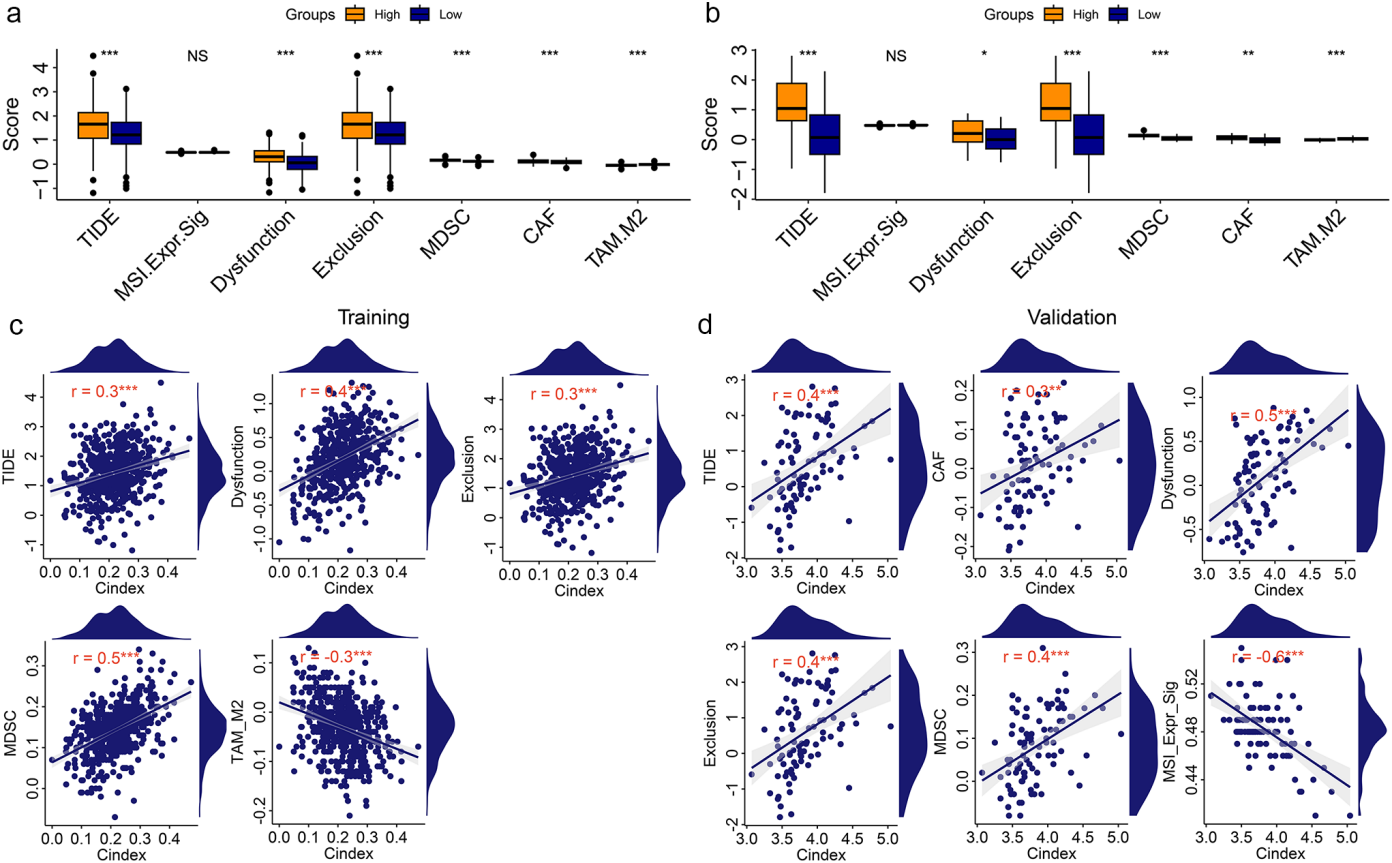
Correlation analysis of risk model with Immunotherapy signatures. **(a, b)** Differentially analysis of immunotherapy-related signatures between high- and low-risk KIRC patients in training group **(a)** and validation group **(b)**. **(c, d)** Point plot of correlation analysis of risk model with immunotherapy signatures in training group **(c)** and validation group **(d)**. *P<0.05. **P<0.01. ***P<0.001. NS, no significance.

Immunotherapy is a new and very promising approach to cancer treatment. Previous studies have found that cuproptosis based treatments can be synergistic with immunotherapy. Therefore, we analyzed the immune status of KIRC patients with different risks. The results showed that there were significant differences between tumor immune microenvironment related signatures scores and immune cell and molecular scores in KIRC patients with different risks (**Supplementary Figure S5**). To further understand the immune status of KIRC patients, we performed a single-cell sequencing analysis using GSE121636 data. We obtained 12 clusters based on genes expression (**Supplementary Figure S6a**). Using the expression of characteristic genes, we annotated these 12 components into four distinct cell types (**Supplementary Figures S6b, c**). The expression of four CRGs (*ATP7A*, *DBT*, *DLST*, and *MTF1*) related to those two biomarkers (LINC02609 and SNHG17) in different types of cells was shown in **Supplementary Figure S6d**. Those four CRGs were mainly expressed in monocyte cell. The correlation of those four type cells were displayed in figure **Supplementary Figure S6e**. These results suggested that the immune status of KIRC patients with different risk patterns may differ. Therefore, to determine whether risk models can guide immunotherapy response, we conducted TIDE based immunotherapy response prediction study. In both the training and validation groups, we found that high-risk KIRC patients had significantly higher TIDE scores than low-risk KIRC patients (**Figures 4a, b**). In addition, several other immunotherapy-related signatures differed significantly between high- and low-risk KIRC patients, including Dysfunction, Exclusion, MDSC, CAF, and TAM_M2 (**Figures 4a, b**). Correlation analyses also showed significant associations between the risk model and multiple immunotherapy-related features (**Figures 4c, d**). However, very coincidentally, we found that the TIDE score and the Exclusion score were consistent in both the TCGA training group and the ICGC validation group.

In addition, oncoPredict algorithm based on GDSC2 was used to explore the response of KIRC patients with different risk score to chemotherapy. Sensitivity to the six drugs varied significantly and consistently between high - and low-risk patients, including Dactolisib_1057, Entospletinib_1630, ERK_2440_1713, ERK_6604_1714, MG-132_1862, and Trametinib_1372 (**Supplementary Figures S7a, b**). The correlation of those drugs with the risk model was showed in **Supplementary Figures S7c, d**.

### 02609 Inhibition of LINCsignificantly reduce the progression of kidney cancer

**Figure 5:**
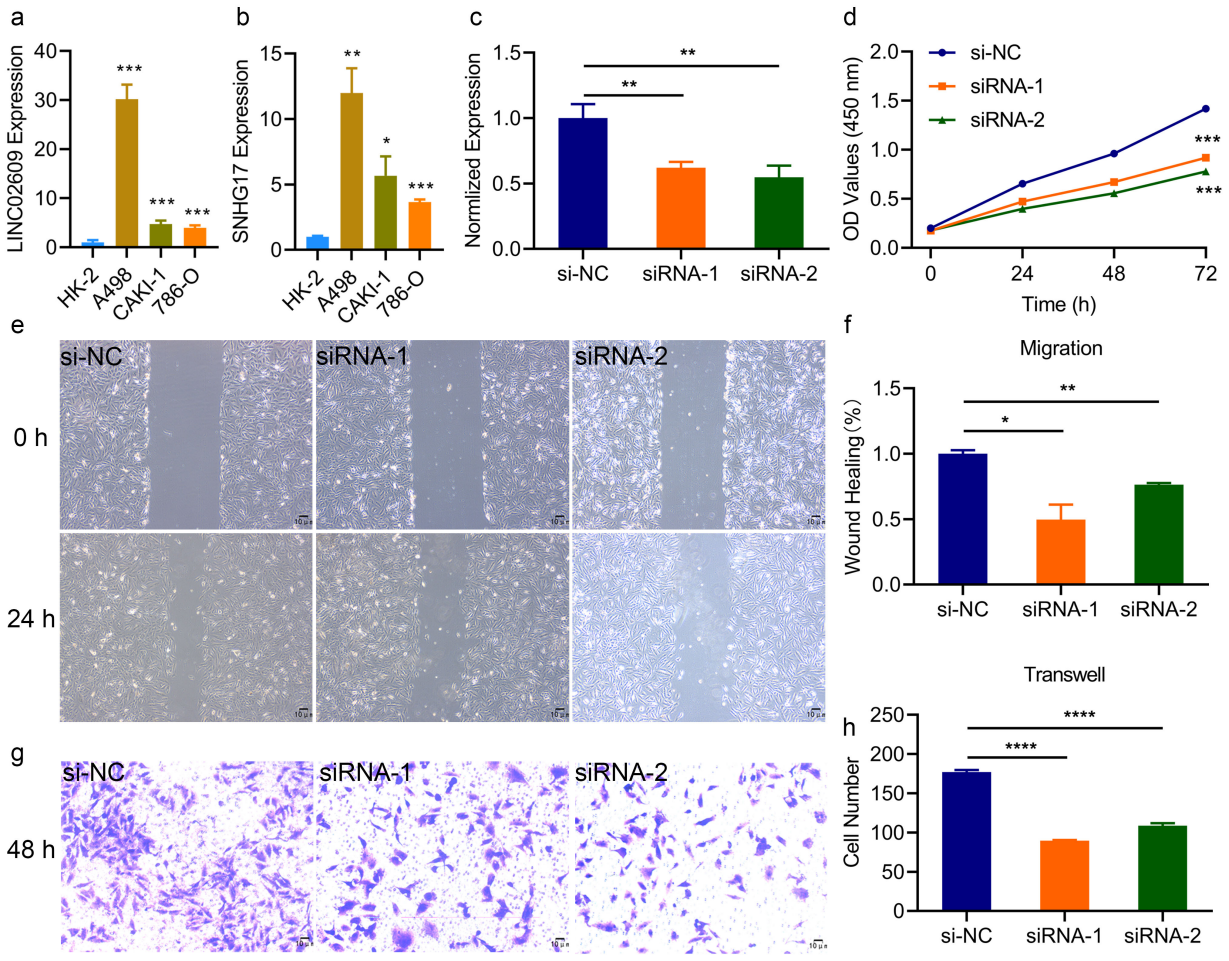
Inhibition of LINC02609 significantly reduce the progression of kidney cancer. **(a, b)** Expression of LINC02609 **(a)** and SNHG17 **(b)** among different cells type. **(c)**, SiRNA of LINC02609 significantly reduced the expression of LINC02609. **(d)**, Cell viability analysis between control and siRNA interference group (n=3). **(e, f)**, Migration ability analysis between control and siRNA interference group. **(e)** migration diagram. **(f)** statistical chart (n=3). The magnification of the picture is 40×. **(g, h)**, Invasion ability analysis between control and siRNA interference group. **(g)** invasion diagram. **(h)** statistical chart (n=3). The magnification of the picture is 100×. *P<0.05. **P<0.01. ***P<0.001. NS, no significance.

These results suggested that LINC02609 and SNHG17 may be prognostic markers of KIRC. To clarify the specific expression of LINC02609 and SNHG17, we first detected their expression in different renal tissue cell lines. Compared with normal tissue cells, LINC02609 and SNHG17 in renal carcinoma cells were significantly elevated (**Figures 5a, b**). Previous studies have found that SNHG17 has a significant increase in KIRC and can promote cancer progression (39, 40). Therefore, we only conducted studies on the role of LINC02609 in the development of KIRC. To clarify the role of LINC02609, we first designed the siRNA of LINC02609. 48 hours after transfection of the siRNA of LINC02609, we collected RNA and performed qPCR detection, and found that the expression of LINC02609 was significantly reduced in the interference groups (**Figure 5c**). Interference with the expression of LINC02609 for 24h, 48h and 72h could significantly reduce the cell viability of A498 cells (**Figure 5d**). The results of wound healing experiment showed that interference with the expression of LINC02609 significantly inhibited the cell migration of A498 compared with the control group (**Figures 5e, f**). Invasion experiments showed that interference with the expression of LINC02609 significantly inhibited the number of A498 cells compared with the control group (**Figures 5g, h**).

### The effect of MAPK signaling pathway agonists in reversing LINC02609

**Figure 6:**
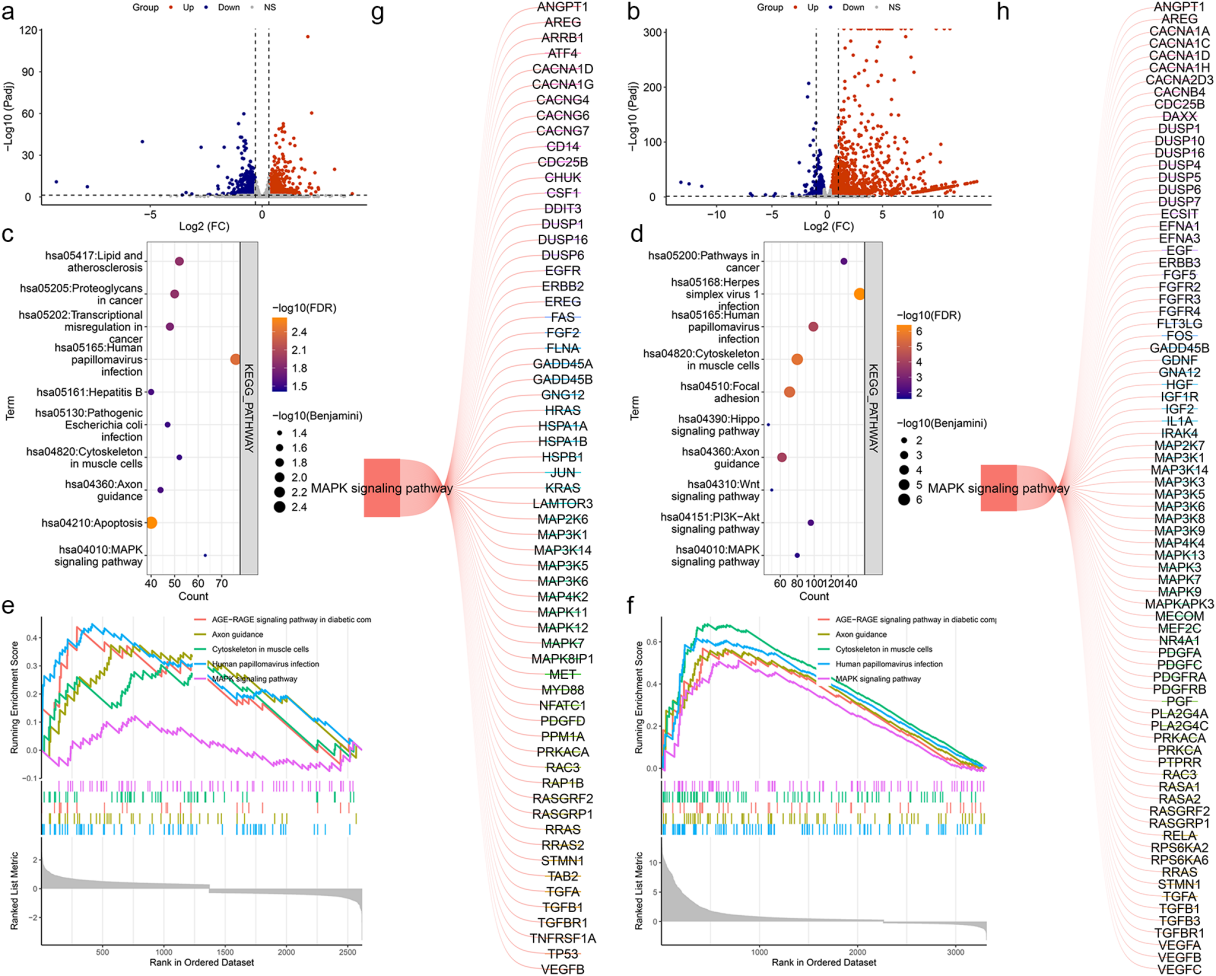
Enrichment analysis based on RNA-seq data by suppressed LINC02609. **(a)** Volcano map of Differentially genes between CTRL and siRNA1. **(b)** Volcano map of Differentially genes between CTRL and siRNA2. **(c)** Bubble plots of the top 10 significantly enriched signal pathways based on the DEGs between CTRL and siRNA1. **(d)** Bubble plots of the top 10 significantly enriched signal pathways based on the DEGs between CTRL and siRNA2. **(e)** The map of the significantly enriched common signal pathways based on the DEGs between CTRL and siRNA1. **(f)** The map of the significantly enriched common signal pathways based on the DEGs between CTRL and siRNA2. **(g)** Differentially expressed genes enriched in the MAPK signaling pathway on the DEGs between CTRL and siRNA1. **(h)** Differentially expressed genes enriched in the MAPK signaling pathway on the DEGs between CTRL and siRNA2.

To clarify the mechanism of LINC0609 in KIRC, we first performed an RNA-seq analysis. The results showed that after interfering with LINC02609 with siRNA1, we found 1477 significantly increased DEGs and 1323 significantly reduced DEGs (**Figure 6a**). After interfering with LINC02609 with siRNA2, we found 2403 significantly increased DEGs and 1158 significantly decreased DEGs (**Figure 6b**). Then, we performed KEGG functional enrichment analysis. The results showed that 26 and 25 KEGG signaling pathways were significantly enriched based on RNA-seq data of siRNA1 and siRNA2 as measured by the Benjamini and FDR values both less than 0.05 (**Supplementary Table S1**). The top 10 signal pathways were represented by **Figure 6c, d**, respectively. The overlap signaling pathways were hsa05165: human papillomavirus infection, hsa04360: axon guidance, hsa04933: AGE-RAGE signaling pathway in complications of diabetes, hsa04820: cytoskeleton of muscle cells, and hsa04010: MAPK signaling pathway (**Figures 6e, f**). The differentially expressed genes enriched in the MAPK signaling pathway are shown in **Figures 6g, h**.

**Figure 7:**
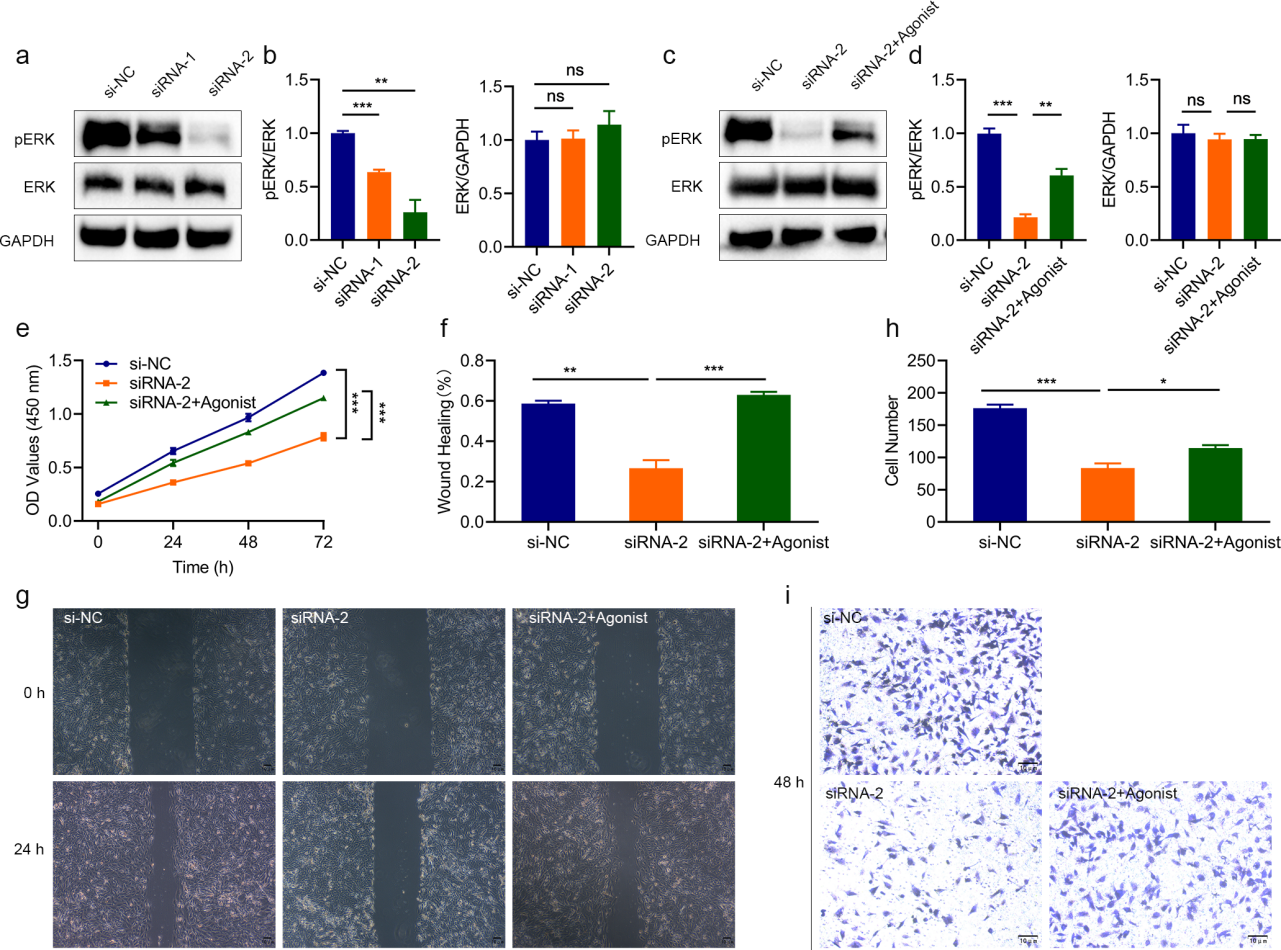
The effect of interfering with LINC02609 was reversed by the MAPK agonist. **(a, b)** Western blot analysis showing that interfering with LINC02609 inhibited the activation of MAPK as measured by the phosphorylation level of ERK. **(c, d)** Western blot analysis showed that interference with LINC02609 could inhibit ERK phosphorylation, which could be reversed by MAPK agonists. **(e)** CCK8 analysis showed that the inhibitory level of cell proliferation by LINC02609 was reversed by the MAPK agonist (n=3). **(f, g)**, Migration analysis showed that the MAPK agonist reversed the inhibitory level of LINC02609 on cell proliferation (n=3). The magnification of the picture is 40×. **(h, i)**, Invasion analysis showed that the MAPK agonist reversed the inhibitory level of LINC02609 on cell proliferation (n=3). The magnification of the picture is 100×. *P<0.05. **P<0.01. ***P<0.001. NS, no significance.

Based on previous studies, to determine whether LINC02609 regulates EOKIRC progression through the MAPK signaling pathway, we then detected the activation level of MAPK signaling pathway, and found that the expression of pERK, a characteristic molecule of the MAPK signaling pathway, was significantly reduced, suggesting that inhibiting LINC02609 could inhibit the activation of the MAPK signaling pathway (**Figures 7a, b**). Next, we conducted rescued studies using agonists of the MAPK signaling pathway (**Figures 7c, d**). The results showed that MAPK agonists could inhibit the proliferation, migration and invasion of cells affected by LINC02609 (**Figure 7e-i**).

## Discussions

KIRC is one of the most common malignant tumors of the genitourinary system (1–4). Even after surgical intervention, the postoperative recurrence rate remains alarmingly high, with a 5-year survival rate of about 10% (5–7). Therefore, identifying patient-specific KIRC prognostic markers and elucidating their functions are critical to improving KIRC outcomes. In this study, TCGA and ICGC datasets were used to screen and validate KIRC prognostic markers. In addition, we conducted *in vitro* functional studies of candidate potential biomarker LINC02609. Through bioinformatics studies, we found that LINC02609 and SNHG17 were significantly elevated in patients with KIRC. This is consistent with previous bioinformatics analysis reports (41–44). To further clarify the expression of LINC02609 and SNHG17, we examined their expression in different renal cell lines and found that LINC02609 and SNHG17 were indeed significantly elevated in renal cancer cell lines. Previous studies have found that SNHG17 expression is significantly elevated in renal cancer, and its high expression is significantly correlated with tumor OS and different clinical features (45–50). We further identified the high expression of SNHG17 in renal carcinoma. Xiao et al. found that LINC02609 was significantly elevated in renal cancer (43). In this study, we found that LINC02609 was significantly elevated in kidney cancer cells which was consistence with previous study (43). Although the high expression of LINC02609 has been verified by experiments, its expression situation still needs to be verified by a large number of clinical samples.

In the study of KIRC prognostic models, many studies have been carried out. Yu et al. used five M6A-associated lncRNAs to construct a risk model, with AUC values of 0.802 and 0.725 in the training group and validation group, respectively (51). Yu et al. used five lncRNAs associated with autophagy to construct a risk model, and the AUC values of the training group and the verification group were 0.81 and 0.71, respectively (52). Cai et al. used 8 immunogenic cell death related models to construct a risk model with an AUC value of 0.75 (53). Ren et al. constructed a risk model using three CCNB2-associated lncRNAs (54). The areas under the total survival ROC curve of stage 1, 3 and 5 were 0.704, 0.702 and 0.741, respectively (54). In this study, we identified two CRDELs independently associated with KIRC OS (LINC02609 and SNHG17) and used them to construct a risk model with an AUC value of 0.70. While the AUC of our model is not the highest, we use relatively few biomarkers. In addition, it is very important that we also carry out validation studies on the model in other independent samples. The AUC value of risk model in the independent sample model is 0.60. Due to the small sample size of other independent samples used in this study. Further sample expansion studies are necessary. Nevertheless, our study further suggests that LINC02609 and SNHG17 may serve as prognostic biomarkers for KIRC (55–58).

SNHG17 is a widely expressed lncRNA in a variety of cancers. Previous studies have found that the expression of SNHG17 is up-regulated in ovarian, gastric, lung, prostate and other cancers (59–62). SNHG17 may promote the development of cancer through various molecular mechanisms, such as up-regulating FOXA1, sponging miR-328-3p, targeting microRNA-375-3p, inhibiting P15 and P16 (45, 59, 63, 64). At present, there are few researches on the role of LINC02609. Xiao et al. found that LncRNA LINC02609 up-regulates the expression of APOL1 in KIRC via sponge miR-149-5p (43). The expression of APOL1 gene inhibited tumor formation, proliferation, metastasis and xenograft tumor formation (43). These results suggested that LINC02609 may be carcinogenic. However, it is a great pity that the authors do not report specific data. In this study, we found that the expression of LINC02609 was significantly elevated. High expression of LINC02609 was significantly associated with poor prognosis of KIRC. Our findings also suggest that LINC02609 may play a role in promoting kidney cancer. Therefore, we conducted a functional study of LINC02609 *in vitro*. We found for the first time that inhibition of LNC02609 significantly reduced the proliferation, migration and invasion of renal carcinoma cells.

The tumor immune microenvironment consists of tumor cells, invasive immune cells, stromal cells and cytokines. Invasive immune cells play an important role in tumor genesis, development and anti-cancer immune regulation, and are a promising therapeutic target. In this study, we found significant differences in immune status between high- and low-risk patients and significant correlation with immunotherapy response-related signatures, further suggesting that cuproptosis may be related to immunotherapy (65, 66). However, it needs to be emphasized that we found that the TIDE score and the Exclusion score are consistent. The TIDE score is calculated by integrating the Exclusion score and the Dysfunction score. If the Dysfunction score of a certain sample is negative (that is, the immune function is not significantly imbalanced), the TIDE score will be directly equal to the Exclusion score. Therefore, we retrospectively tested the immunotherapy response data and found that there was indeed a phenomenon where the Dysfunction score of some KIRC patients was negative. But why are the TIDE scores and Exclusion scores of other KIRC patients the same? Although KIRC is usually dominated by T-cell infiltration and Dysfunction, certain specific subtypes (such as highly fibrotic tumors) may mediate immune Exclusion through the stromal barrier. This leads to the TIDE score being dominated by the Exclusion mechanism (67–70). Therefore, we retrospectively analyzed the fibroblast activation marker COL1A1 and found that COL1A1 was indeed significantly highly expressed, which supported the above hypothesis (71, 72). The calculation of TIDE score relies on the preset gene set and standardized methods, and the universality of the results needs to be cross-verified in other algorithms. Overall, this study reports the consistency phenomenon between TIDE score and Exclusion score in KIRC, suggesting that it may reflect a unique type of immune escape. In the future, multi-omics data and clinical intervention trials need to be combined to reveal the biological basis and therapeutic significance of this phenomenon. If some KIRC are indeed dominated by immune Exclusion, traditional immune checkpoint inhibitors (such as anti-PD-1) may have limited efficacy, and drugs targeting the stromal barrier (such as anti-TGF-β or anti-CTGF) need to be combined to enhance T cell infiltration (73, 74). Furthermore, the oncoPredict algorithm showed that the sensitivity of six drugs was associated with risk model. The effectiveness of these drugs in other cancers indicates that they may be regarded as a treatment for kidney cancer, and further confirms that copper nephropathy is related to the sensitivity to chemotherapy drugs (75).

Our research results reveal the key carcinogenic role of LINC02609 in the progression of KIRC, mediated by its regulatory effect on the MAPK signaling pathway. Silencing LINC02609 can significantly weaken the activation of the MAPK pathway, as evidenced by the decreased phosphorylation of ERK1/2, which is associated with the reduced proliferation, migration and invasion abilities of KIRC cells. It is worth noting that the application of MAPK agonists effectively rescued these phenotypic changes, confirming that LINC02609-driven malignant tumors are mechanistically dependent on MAPK signaling. This is consistent with the established role of overactive MAPK signaling in promoting tumorigenesis in various cancer types, including KIRC, by facilitating cell cycle progression, epithelial-mesenchymal transition (EMT), and extracellular matrix remodeling (76).

Although our research suggests that LINC026089 achieves its carcinogenic effect through the MAPK signaling pathway. But there are also some shortcomings in our research. The exact mechanism by which LINC02609 regulates the MAPK signal remains to be further studied. As a long non-coding RNA (lncRNA), LINC02609 may act as a molecular scaffold or chromatin remodeler to promote the assembly of the MAPK signaling complex. For example, it is known that lncRNAs such as MALAT1 and HOTAIR can interact with components of the MAPK pathway (such as Raf kinase or ERK), or regulate the transcription of the upstream receptor tyrosine kinase of MAPK (77, 78). Therefore, how inhibiting LINC02609 achieves the inhibition of the MAPK signaling pathway is highly worthy of subsequent research.

Clinically, targeting LINC02609 is a promising therapeutic strategy for KIRC, especially considering the limitations of directly using MAPK inhibitors to treat this malignant tumor. Although MAPK-targeted therapies (such as MEK inhibitors) have shown efficacy in melanoma and lung cancer, their application in KIRC is hindered by compensatory feedback loops and dose-limiting toxicity (76). In contrast, silencing LINC02609 can weaken the MAPK signal of the proximal node, potentially bypassing the resistance mechanism. However, there are still some challenges. Firstly, the tissue specificity and off-target effects of LINC02609 targeting need to be strictly evaluated in preclinical models. Secondly, in the KIRC patient cohort, the correlations between the expression level of LINC02609, MAPK activity and clinical outcomes need to be verified. Finally, identifying small molecule inhibitors that can selectively inhibit LINC02609 or RNA-based therapies will be crucial for translation applications.

## Conclusion

In this study, we found that LINC02609 and SNHG17 were significantly elevated in patients with KIRC and were independently associated with the survival status of KIRC. The risk model based on LINC02609 and SNHG17 can predict prognosis well and guide the selection of clinical treatment regimens. *In vitro* experiments have shown that the inhibitory effect of interfering with LINC02609 on renal cell carcinoma is reversed by MAPK agonists, suggesting that LINC02609 may achieve its pro-cancer effect through the MAPK signaling pathway. Although the risk model was verified by another external independent sample, due to the small sample size of the external independent sample, the risk model still needs further verification. Although the function of LINC02609 has been verified *in vitro*, further *in vivo* verification studies are still needed.

## Data Availability

The original contributions presented in the study are included in the article/**Supplementary Material**. Further inquiries can be directed to the corresponding author/s.
