# Supplementary material for: Correlation study of LINC02609 and SNHG17 as prognostic biomarkers of kidney renal clear cell carcinoma and therapeutic sensitivity based on public data and In Vitro analysis
Source: Front Immunol. 2025 May 26;16:1592474. doi: 10.3389/fimmu.2025.1592474 (PMC12146389; doi:10.3389/fimmu.2025.1592474)
Supplement: Supplementary file 1 [file DataSheet1.pdf]

Correlation Study of LINC02609 and SNHG17 as Prognostic Biomarkers of Kidney renal clear cell carcinoma and Therapeutic Sensitivity Based on Public Data and *In Vitro* Analysis

Chaoqun Xing[1#], Weiwei Zou[2#], Yangqin Li[1#], Ti Zhang[1], Fan Yao[1], Zhi-Yong Yao[1], Xiao-Liang Xing[1*]

[1]The First Affiliated Hospital of Hunan Medical University, School of Public Health and Emergency Response, Hunan University of Medicine, Huaihua 418000, Hunan, P. R. China.

[2]The Second People's Hospital of Huaihua, Huaihua 418000, Hunan, P. R. China.

[#]Contributes equally to this work.

[*]Correspondence author: Xiao-Liang Xing, xiaoliangxinghnm@126.com

Supplementary information: Seven figures and one table.

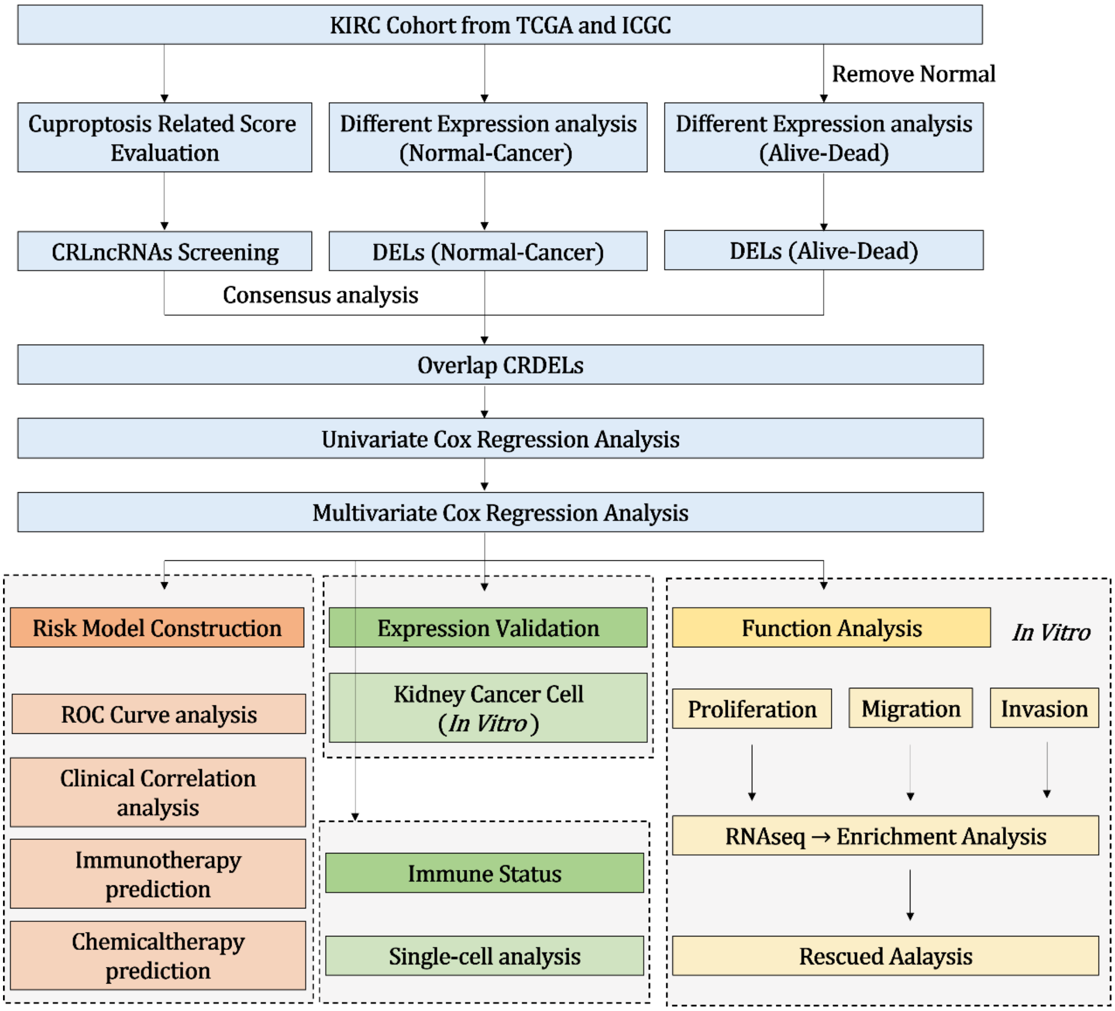

Supplementary Figure 1. The flow chart in Supplementary Figure 1 depicted the data analysis process.

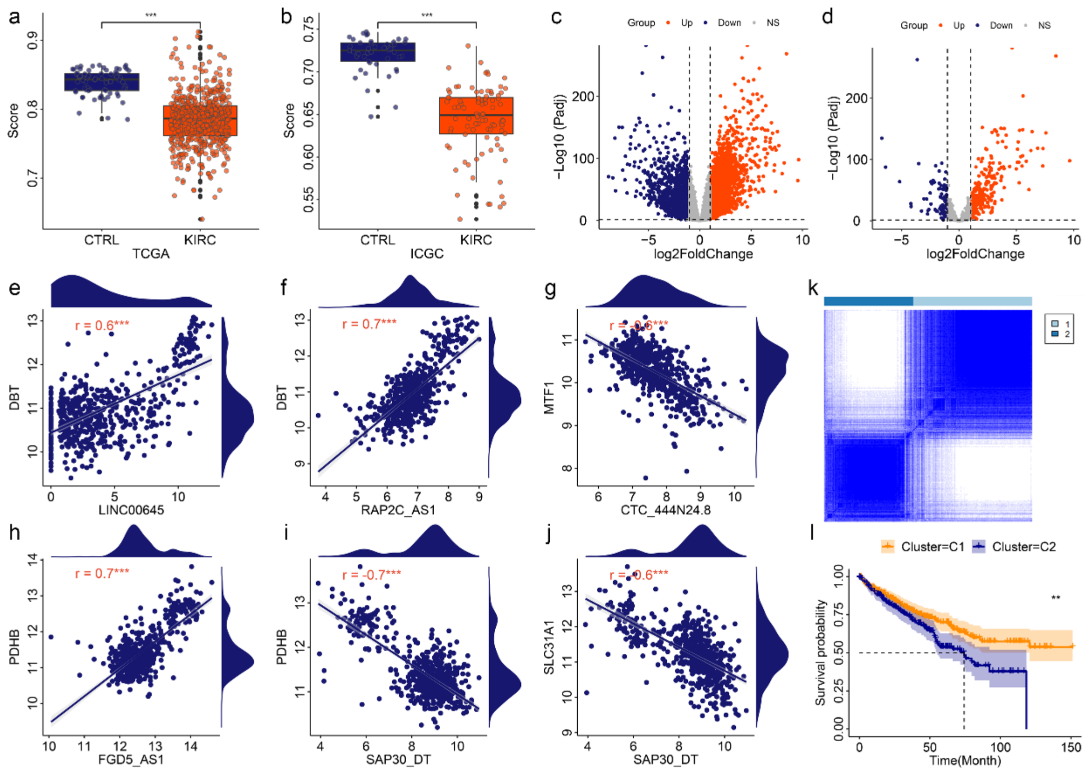

Supplementary Figure 2. Screening and consistency analysis of CRDELs.

(a-b) Difference analysis of cuproptosis score between normal and KIRC patients in training group (a) and validation group (b). Volcanic maps of DEGs (c) and DELs (d) in training group. (e-g) The top three positive correlation between cuptoptosis related genes and DELs. (h-j) The top three negative correlation between cuptoptosis related genes and DELs. (k) Classification cluster analysis diagram based on CRDELs gene. (l) K-M curve of 2 classification cluster. *, P<0.05. **, P<0.01. ***, P<0.001.

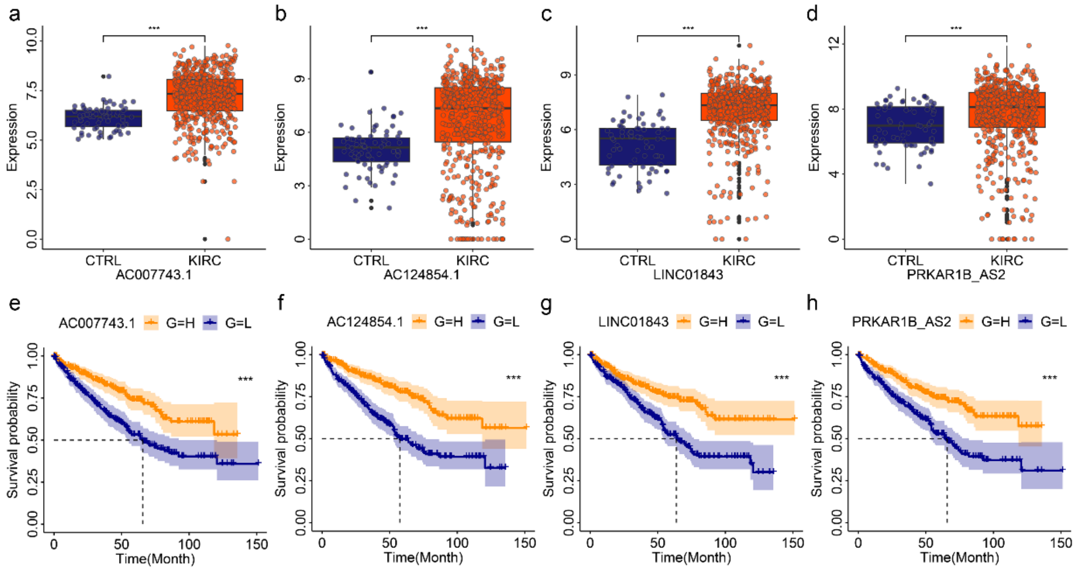

Supplementary Figure 3. Four signature representations that contradict each other.

(a-d) Expression of AC007743.1 (a), AC124854.1 (b), LINC01843 (c), and PRKAR1B-AS2 (d) between normal and KIRC patients. (e-h) K-M curve of AC007743.1 (e), AC124854.1 (f), LINC01843 (g), and PRKAR1B-AS2 (h). *, P<0.05. **, P<0.01. ***, P<0.001.

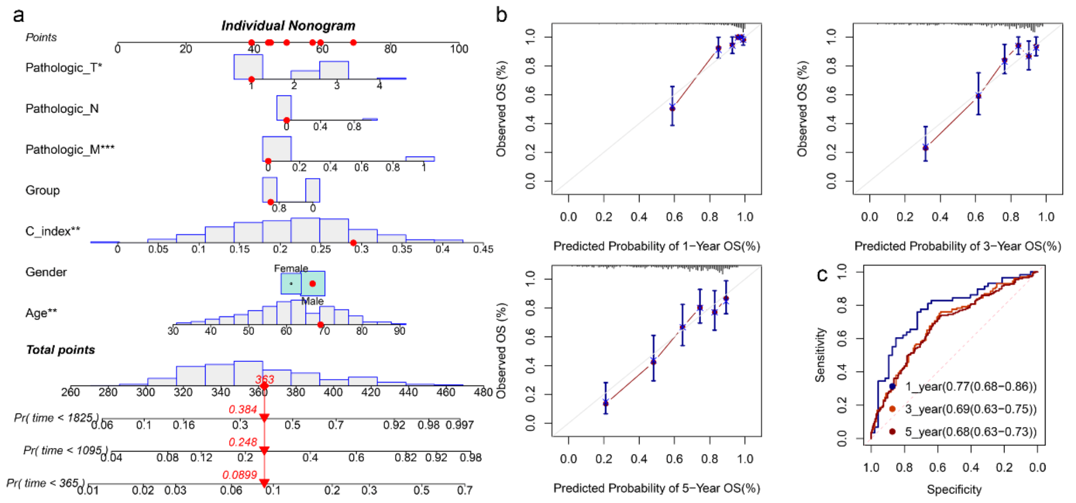

Supplementary Figure 4. Nomogram analysis of risk model in entire group.

(a) The Nomograms were used to predict 1-, 3-, and 5-year survival rates of KIRC patients. (b) The calibration curves of the nomogram at 1-, 3-, and 5- years. (c), Time dependent ROC curve of risk model.

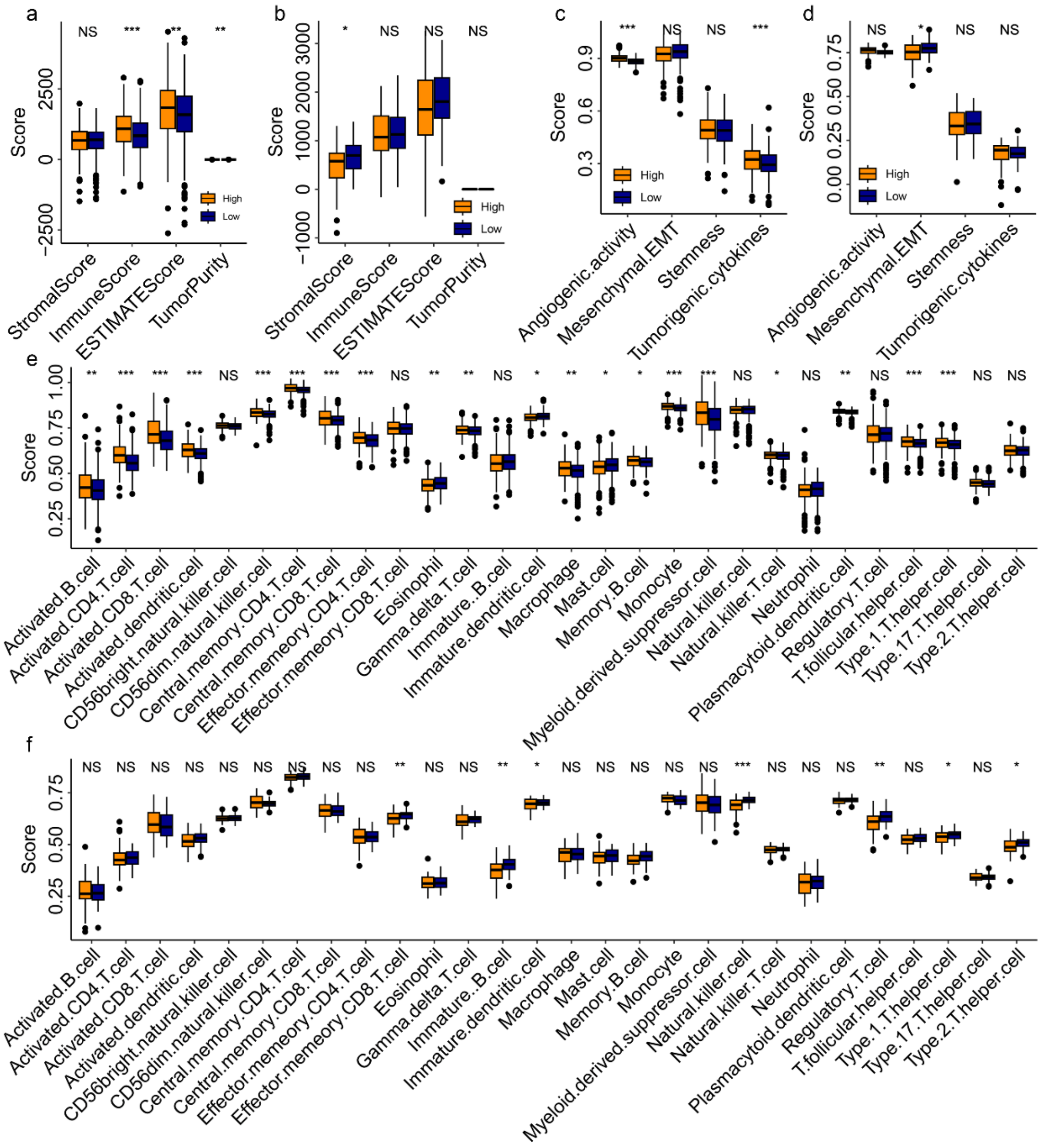

Supplementary Figure 5. Immune landscape analysis in different risk groups.

(a-b) Difference analysis for tumor immune microenvironment in training (a) and validation (b) groups. (c-d) Difference analysis for immune factors in training (c) and validation (d) groups. (e-f) Difference analysis for immune cells in training (e) and validation (f) groups.

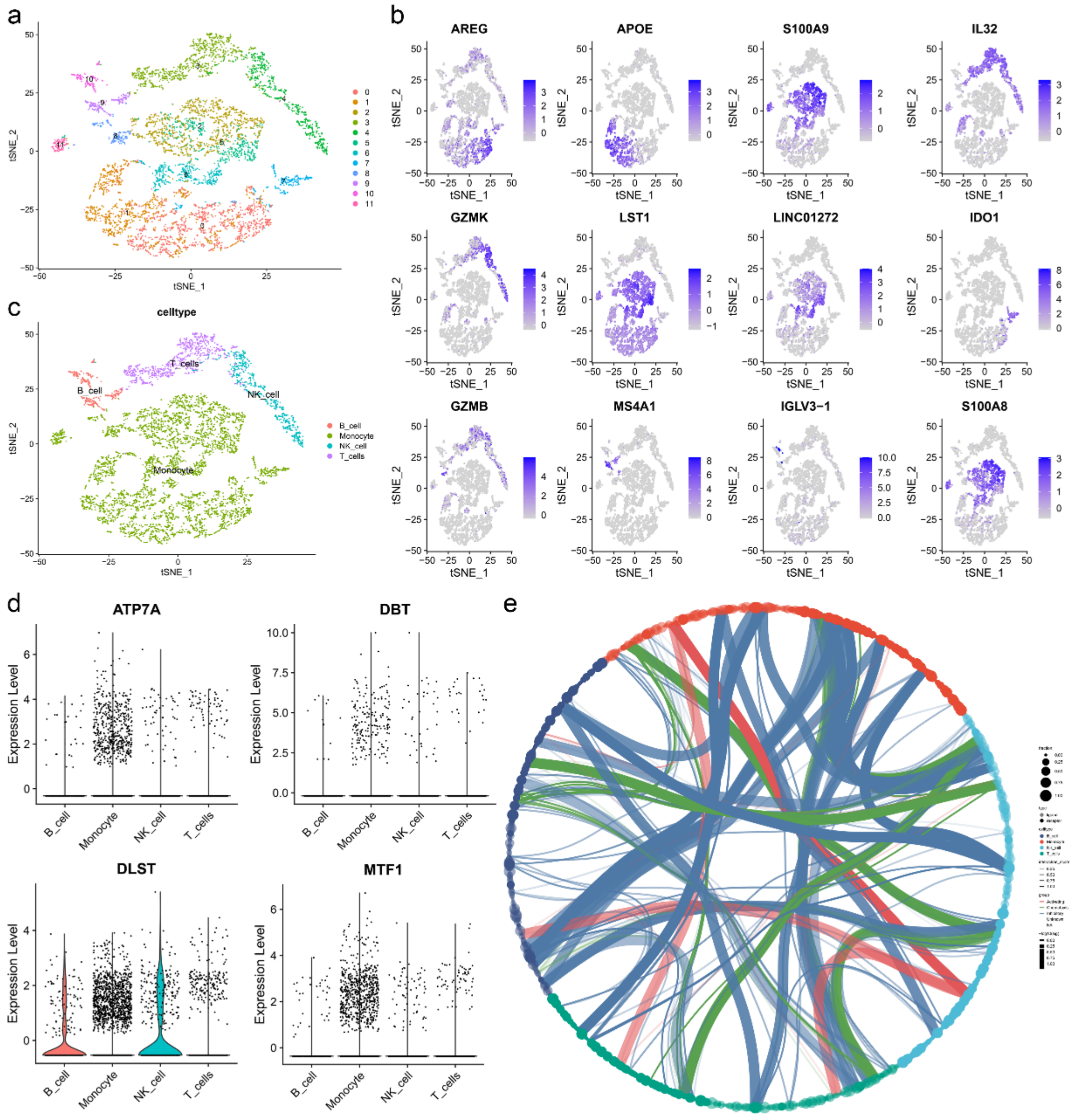

Supplementary Figure 6. Single cell RNA-seq analysis.

(a) Seurat_cluster analysis based on 0.2 resolution. (b) Feature plots of 12 seurat_cluster. (c) tSNE distribution in four cell types. (d) Expression of four CRGs in four cell types. (e) Communication of four cell type.

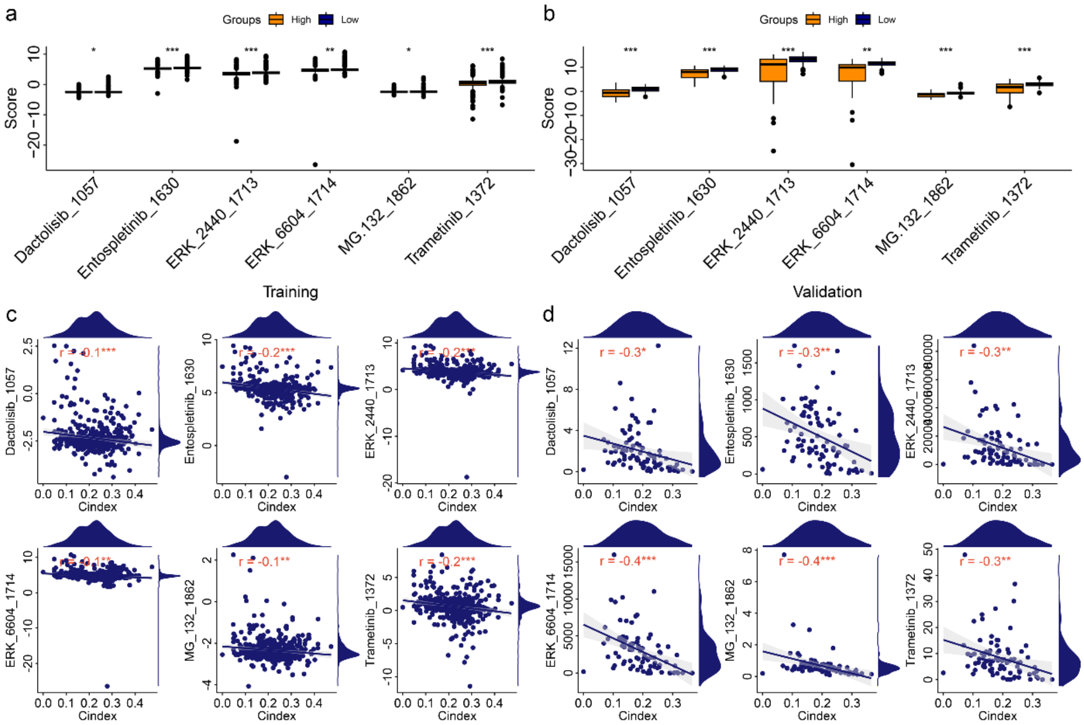

Supplementary Figure 7. Correlation analysis of risk model with chemotherapy drugs. (a-b) Differentially analysis of chemotherapy drugs between high- and low-risk KIRC patients in training group (a) and validation group (b). (c-d) Point plot of correlation analysis of risk model with chemotherapy drugs in training group (c) and validation group (d). *, P<0.05. **, P<0.01. ***, P<0.001. NS, no significance.

Supplementary Table 1. The KEGG pathway was significantly enriched

	Term	Count	PValue	Benjamini	FDR
**CTRL vs siRNA1**	hsa05168:Herpes simplex virus 1 infection	154	0.0000	0.0000	0.0000
	hsa04820:Cytoskeleton in muscle cells	80	0.0000	0.0000	0.0000
	hsa04512:ECM-receptor interaction	40	0.0000	0.0000	0.0000
	hsa04510:Focal adhesion	71	0.0000	0.0000	0.0000
	hsa05165:Human papillomavirus infection	99	0.0000	0.0001	0.0001
	hsa04360:Axon guidance	62	0.0000	0.0001	0.0001
	hsa05200:Pathways in cancer	135	0.0001	0.0043	0.0039
	hsa04933:AGE-RAGE signaling pathway in diabetic complications	36	0.0001	0.0043	0.0039
	hsa05412:Arrhythmogenic right ventricular cardiomyopathy	32	0.0001	0.0043	0.0039
	hsa05410:Hypertrophic cardiomyopathy	35	0.0002	0.0055	0.0050
	hsa04151:PI3K-Akt signaling pathway	96	0.0002	0.0067	0.0061
	hsa05414:Dilated cardiomyopathy	36	0.0002	0.0072	0.0066
	hsa04010:MAPK signaling pathway	80	0.0006	0.0170	0.0155
	hsa05418:Fluid shear stress and atherosclerosis	43	0.0009	0.0233	0.0213
	hsa04610:Complement and coagulation cascades	30	0.0010	0.0233	0.0213
	hsa00514:Other types of O-glycan biosynthesis	19	0.0013	0.0282	0.0258
	hsa04310:Wnt signaling pathway	50	0.0015	0.0282	0.0258
	hsa04390:Hippo signaling pathway	46	0.0015	0.0282	0.0258
	hsa00100:Steroid biosynthesis	11	0.0015	0.0282	0.0258
	hsa04540:Gap junction	30	0.0022	0.0371	0.0339
	hsa01521:EGFR tyrosine kinase inhibitor resistance	27	0.0022	0.0371	0.0339
	hsa04072:Phospholipase D signaling pathway	43	0.0030	0.0436	0.0399
	hsa04668:TNF signaling pathway	36	0.0030	0.0436	0.0399
	hsa04142:Lysosome	39	0.0031	0.0436	0.0399
	hsa04514:Cell adhesion molecules	45	0.0031	0.0436	0.0399
**CTRL vs**	hsa04115:p53 signaling pathway	28	0.0000	0.0011	0.0010

**siRNA2**	hsa04210:Apoptosis	40	0.0000	0.0027	0.0023
	hsa05219:Bladder cancer	18	0.0000	0.0030	0.0026
	hsa05165:Human papillomavirus infection	76	0.0001	0.0047	0.0040
	hsa05205:Proteoglycans in cancer	50	0.0002	0.0145	0.0126
	hsa05417:Lipid and atherosclerosis	52	0.0002	0.0145	0.0126
	hsa05202:Transcriptional misregulation in cancer	48	0.0004	0.0189	0.0164
	hsa05134:Legionellosis	19	0.0007	0.0300	0.0261
	hsa04360:Axon guidance	44	0.0009	0.0320	0.0278
	hsa05161:Hepatitis B	40	0.0010	0.0320	0.0278
	hsa04933:AGE-RAGE signaling pathway in diabetic complications	28	0.0010	0.0320	0.0278
	hsa05130:Pathogenic Escherichia coli infection	47	0.0012	0.0352	0.0306
	hsa05223:Non-small cell lung cancer	22	0.0013	0.0352	0.0306
	hsa04820:Cytoskeleton in muscle cells	52	0.0014	0.0358	0.0311
	hsa04218:Cellular senescence	38	0.0017	0.0407	0.0353
	hsa04710:Circadian rhythm	13	0.0022	0.0447	0.0388
	hsa04071:Sphingolipid signaling pathway	31	0.0023	0.0447	0.0388
	hsa04010:MAPK signaling pathway	63	0.0023	0.0447	0.0388
	hsa05210:Colorectal cancer	24	0.0027	0.0447	0.0388
	hsa05212:Pancreatic cancer	22	0.0027	0.0447	0.0388
	hsa05220:Chronic myeloid leukemia	22	0.0027	0.0447	0.0388
	hsa05222:Small cell lung cancer	25	0.0031	0.0447	0.0388
	hsa00562:Inositol phosphate metabolism	21	0.0032	0.0447	0.0388
	hsa05218:Melanoma	21	0.0032	0.0447	0.0388
	hsa03083:Polycomb repressive complex	23	0.0032	0.0447	0.0388
	hsa05213:Endometrial cancer	18	0.0036	0.0483	0.0419
